# Reduction of estimated glomerular filtration rate after COVID-19-associated acute kidney injury

**DOI:** 10.1590/2175-8239-JBN-2022-0179en

**Published:** 2023-06-09

**Authors:** Gabrielle Accioly Omena Bento, Vivian Larissa Tenório Leite, Rodrigo Peixoto Campos, Flora Braga Vaz, Elizabeth De Francesco Daher, Daniella Bezerra Duarte

**Affiliations:** 1 Centro Universitário Tiradentes, Faculdade de Medicina, Maceió, AL, Brazil.; 2 Universidade Federal de Alagoas, Faculdade de Medicina, Maceió, AL, Brazil.; 3 Centro Universitário CESMAC, Faculdade de Medicina, Maceió, AL, Brazil.; 4 Universidade Federal do Ceará, Faculdade de Medicina, Fortaleza, CE, Brazil.

**Keywords:** COVID-19, Renal Insufficiency, Chronic, Acute Kidney Injury, Glomerular Filtration Rate, COVID-19, Insuficiência Renal Crônica, Injúria Renal Aguda, Taxa de Filtração Glomerular

## Abstract

**Introduction:**

Acute Kidney Injury (AKI), a frequent manifestation in COVID-19, can compromise kidney function in the long term. We evaluated renal function after hospital discharge of patients who developed AKI associated with COVID-19.

**Methods:**

This is an ambidirectional cohort. eGFR and microalbuminuria were reassessed after hospital discharge (T1) in patients who developed AKI due to COVID-19, comparing the values with hospitalization data (T0). P < 0.05 was considered statistically significant.

**Results:**

After an average of 16.3 ± 3.5 months, 20 patients were reassessed. There was a median reduction of 11.5 (IQR: –21; –2.1) mL/min/1.73m^2^ per year in eGFR. Forty-five percent of patients had CKD at T1, were older, and had been hospitalized longer; this correlated negatively with eGFR at T1. Microalbuminuria was positively correlated with CRP at T0 and with a drop in eGFR, as well as eGFR at admission with eGFR at T1.

**Conclusion:**

There was a significant reduction in eGFR after AKI due to COVID-19, being associated with age, length of hospital stay, CRP, and need for hemodialysis.

## RESUMO

**Introdução:**

A Injúria Renal Aguda (IRA), uma manifestação frequente na COVID-19, pode comprometer a função renal em longo prazo. Avaliamos a função renal após a alta hospitalar de pacientes que desenvolveram IRA associada à COVID-19.

**Métodos:**

Esta é uma coorte ambidirecional. A TFGe e a microalbuminúria foram reavaliadas após a alta hospitalar (T1) em pacientes que desenvolveram IRA devido à COVID-19, comparando os valores com dados de hospitalização (T0). P < 0,05 foi considerado estatisticamente significativo.

**Resultados:**

Após uma média de 16,3 ± 3,5 meses, 20 pacientes foram reavaliados. Houve uma redução média de 11,5 (IIQ: –21; –2,1) mL/min/1,73m2 por ano na TFGe. Quarenta e cinco por cento dos pacientes apresentaram DRC no T1, eram mais velhos e haviam sido hospitalizados por mais tempo; isso se correlacionou negativamente com a TFGe no T1. A microalbuminúria foi positivamente correlacionada com a PCR no T0 e com uma queda na TFGe, assim como a TFGe na admissão com a TFGe no T1.

**Conclusão:**

Houve uma redução significativa na TFGe após IRA devido à COVID-19, sendo associada à idade, tempo de internação, PCR e necessidade de hemodiálise.

## Introduction

In addition to the respiratory system, COVID-19 affects other systems, such as the kidney, leading to Acute Kidney Injury (AKI). The pathogenesis of COVID-19-associated AKI involves direct virus injury to the kidney and indirect injury by cytokine storm, hypoxia, and hypoperfusion^1,2^.

The incidence of AKI due to COVID-19 can range from 4.7% to 55.9%, and AKI is associated with a higher mortality compared to patients without AKI^3,4,5^. About 32% of individuals with AKI associated with COVID-19 may not fully recover their kidney function after hospital discharge^6^.

Progression to CKD after AKI may be due to failure to recover from AKI, development of CKD after recovery from AKI, or progression of pre-existing CKD^7^. A longitudinal cohort found that 35% of patients who develop AKI associated with COVID-19 had reduced glomerular filtration rate (GFR) six months after hospital discharge^8^.

To date, there are no Brazilian data on the evolution of renal function in patients with AKI associated with COVID-19. Therefore, the objective of this study was to evaluate renal function after hospital discharge in patients who developed AKI associated with COVID-19.

## Methods

This was an ambidirectional cohort. The following data were collected from the medical records of patients hospitalized at Hospital Santa Casa de Misericórdia de Maceió (HSCMM) with COVID-19 who had AKI between March 2020 and May 2021 (T0): age, gender, length of hospital stay, comorbidities such as systemic arterial hypertension (SAH) and diabetes mellitus (DM), use of iACE or ARB, AKI stages according to AKIN, need for hemodialysis (HD), laboratory dosages (creatinine, urea, C-reactive protein – CRP, ferritin, lymphocyte count), pulmonary involvement of COVID-19 on chest computed tomography (CT)), need for invasive mechanical ventilation (IMV), and use of vasoactive amines. In a second moment (T1), the patients were reassessed and submitted to anamnesis, measurement of blood pressure (BP), and dosage of creatinine, microalbuminuria and urine summary. Creatinine values were used to estimate GFR using the CKD-EPI (2021) equation (eGFR). Sarcopenia was an exclusion criterion. Data were analyzed using IBM SPSS Statistics software for Macintosh (Version 23.0. Armonk, NY: IBM Corp). Categorical variables are presented as absolute count and relative frequency in percentages. To assess the association between categorical variables, the chi-square or Fisher’s exact tests were used. Continuous variables were evaluated for normality using the Shapiro-Wilk test, and Q-Q plots, histograms, and dispersion measures. Variables with normal distribution are presented as mean ± standard deviation and those with non-normal distribution as median and interquartile range (IQR). Student’s t-test and Mann-Whitney test were used for independent comparisons of normal and non-normal data, respectively. To compare two dependent groups, the paired t-test was used. Correlation analyses between continuous variables were performed using Spearman’s non-parametric correlation with rho coefficient. P values < 0.05 were considered significant. The study was approved by the National Research Ethics Committee (CAAE 51031521.3.0000.5641) and patients gave their consent to participate in the study.

## Results

**Figure 1.**
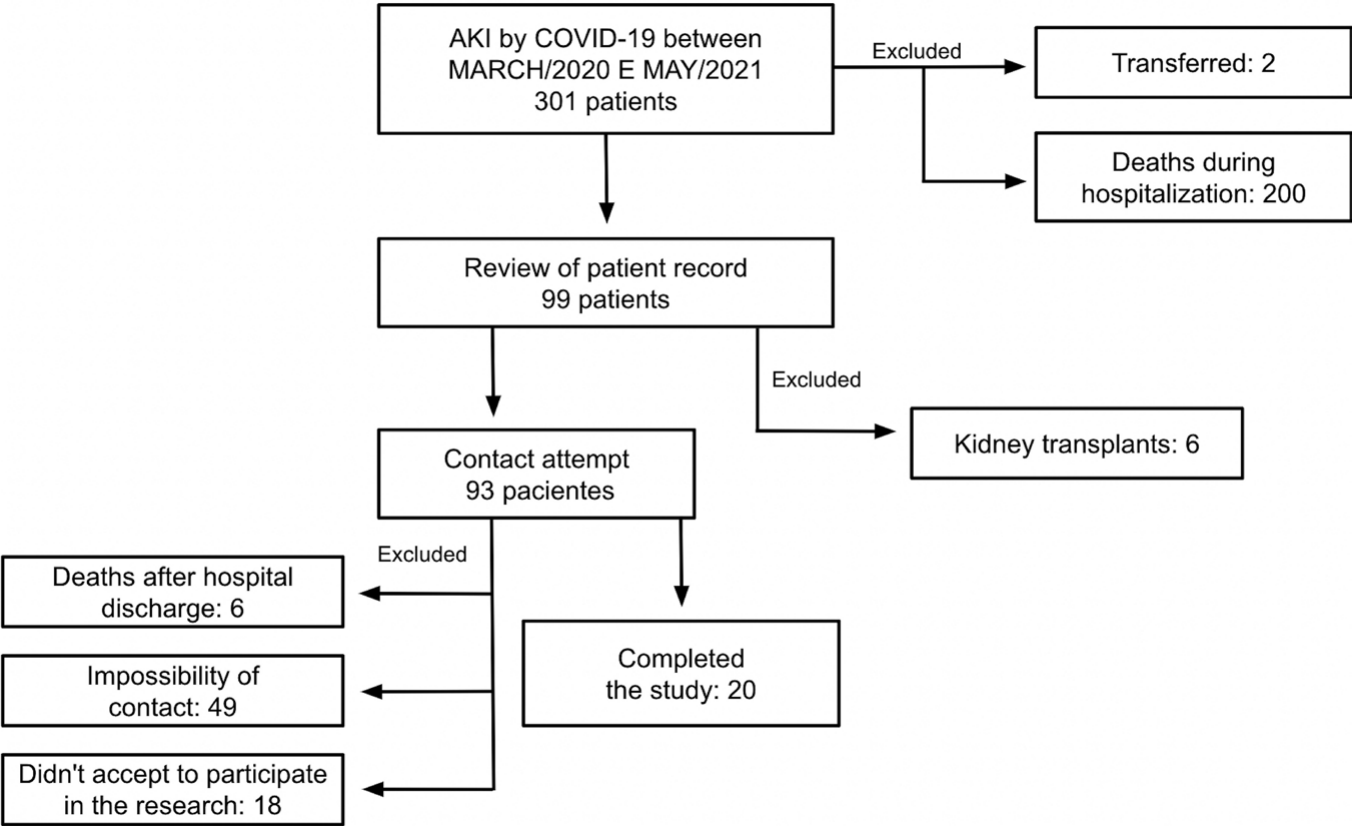
Selection of patients and reasons for exclusion from the study. AKI: acute kidney injury.

In total, 301 patients with COVID-19 were evaluated, of which 200 (61.7%) died and 2 were transferred, leaving 99 survivors, of which only 20 were reassessed (Figure 1). The mean age of patients at hospital discharge was 61.9 ± 11.3 years and 50% were female; 65% were hypertensive and 25% had diabetes. The median length of hospital stay was 45 days (IQR: 25; 50 days). Baseline creatinine (Cr) had a median of 0.78 (IQR: 0.62; 0.9 mg/dL) and mean baseline eGFR was 95.9 ± 16.7 mL/min/1.73m^2^, with a median Cr at hospital discharge of 1.25 (IQR: 0.88; 1.83 mg/dL) (Table 1). Only one patient (5%) did not require ICU admission.

**Table 1.** Summary of clinical and laboratory data of patients in T0 and T1 and according to the need for hemodialysis during hospitalization

	Total group (n = 20)	No Hemodialysis (n = 9)	Hemodialysis (n = 11)	p
**Data at hospital admission (T0)**				
***Age, years***	62 ± 11	60 ± 14	64 ± 8	0.151
***Female gender, n (%)***	10 (50)	3 (33.3)	7 (63.6)	0.37
***Hospitalization time, days***	45 (25; 50)	42 (18; 46)	47 (35; 52)	0.37
***Comorbidities and treatments***				
Diabetes mellitus, n (%)	5 (25)	2 (22.2)	3 (27.3)	1.000
Arterial hypertension, n (%)	13 (65)	6 (66.7)	7 (63.6)	1.000
Use of ACE inhibitors or ARBs, n (%)	8 (40)	3 (33.3)	5 (45.5)	0.67
***Laboratory data and Kidney function***				
CRP on admission, mg/dL	16.7 (3.1; 23)	4.1 (2.5; 20.4)	19.5 (4.3; 23.5)	0.175
Ferritin on admission, µg/L	1057 (532; 1521)	1140 (1029; 1636)	641 (399; 1136)	0.257
Lymphocyte count on admission, /mm^3^	1219 ± 449	1111 ± 568	1307 ± 325	0.376
Basal serum Creatinine, mg/dL	0.78 (0.62; 0.9)	0.8 (0.72; 0.9)	0.7 (0.58; 0.96)	0.456
Basal eGFR, mL/min/1.73m^2^	95.9 ± 16.7	101.4 ± 14.2	91.3 ± 17.8	0.183
Serum creatinine on admission, mg/dL	0.99 (0.78; 1.1)	1.1 (0.98; 1.5)	0.84 (0.61; 1)	0.031
Blood urea on admission, mg/dL	33 (28; 55)	43 (31; 50)	33 (23; 62)	0.261
eGFR on admission, mL/min/1.73m^2^	81 ± 24	73 ± 28	88 ± 18	0.16
AKIN, n (%)				0.006
*Stage 1*	2 (10)	2 (22.2)	0 (0)	
*Stage 2*	3 (15)	3 (33.3)	0 (0)	
*Stage 3*	15 (75)	4 (44.4)	11 (100)	
***Parameters during hospital stay***				
Maximum CRP, mg/dL	32.9 (27.8; 43.9)	30.5 (25.7; 33.2)	36.9 (31.3; 47.6)	0.201
Maximum ferritin, µg/L	1650 (1504; 2982)	1901 (1650; 4029)	1597 (1417; 1650)	0.142
Minimum lymphocyte count, /mm^3^	450 ± 292	420 ± 236	474 ± 340	0.691
Maximum serum creatinine, mg/dL	3.89 (3.17; 6.12)	2.94 (1.73; 3.78)	6.1 (3.94; 7.71)	<0.001
Maximum blood urea, mg/dL	200 (129; 238)	117 (85; 153)	224 (210; 257)	0.001
Serum creatinine on hospital discharge, mg/dL	1.25 (0.88; 1.83)	1.2 (0.85; 1.4)	1.5 (0.89; 2.35)	0.201
***Pulmonary function during hospital stay***				
Pulmonary involvement on CT, n (%)				0.564
*<25%*	6 (33.3)	4 (44.4)	2 (22.2)	
*25% – 50%*	3 (16.7)	2 (22.2)	1 (11.1)	
*50% – 75%*	6 (33.3)	2 (22.2)	4 (44.4)	
*>75%*	3 (16.7)	1 (11.1)	2 (22.2)	
Invasive ventilation, n (%)	15 (75)	5 (55.6)	10 (90.9)	0.127
Use of Vasopressor, n (%)	17 (85)	6 (66.7)	11 (100)	0.074
**Reassessment period (T1)**				
Time from discharge to follow-up, months	16 ± 3	15 ± 4	17 ± 3	0.329
Systolic blood pressure, mmHg	144 ± 17	147 ± 22	142 ± 11	0.537
Diastolic blood pressure, mmHg	93 ± 16	94 ± 22	92 ± 10	0.794
Serum creatinine, mg/dL	1.19 (0.95; 1.41)	1.06 (0.96; 1.26)	1.2 (0.79; 1.56)	0.71
eGFR, mL/min/1.73 m^2^	66 ± 22	71 ± 22	62 ± 22	0.386
Albuminuria, mg/g	8.3 (6; 37)	6.8 (6; 13.6)	10 (6; 41.4)	0.497
eGFR reduction, mL/min/1.73 m^2^	–16 (–30; –3)	–5 (–10; 7)	–19 (–39; –17)	0.004
eGFR reduction, %	–18.5 (–33; –2.5)	–4 (–14; 8)	–28 (–43; –18)	0.006
eGFR reduction rate, mL/min/year	–11.5 (–21; –2.1)	–3.5 (–10.9; –6)	–17 (–24; –9.9)	0.016
eGFR < 60 mL/min/1.73 m^2^	9 (45)	3 (33.3)	6 (54.5)	0.406
Microalbuminuria, n (%)	6 (31.6)	2 (22.2)	4 (40)	0.628
Hematuria, n (%)	4 (20)	1 (11.1)	3 (27.3)	0.591

ACEI: angiotensin-converting enzyme inhibitor, ARB: angiotensin receptor blocker, Cr: creatinine, eGFR: estimated glomerular filtration rate, AKIN: Acute Kidney Injury Network, Ur: urea, CRP: C-reactive protein, CT: computed tomography.

During hospitalization, there were 2 patients (10%) in stage 1 AKI, 3 (15%) in stage 2, and 15 (75%) in stage 3 according to AKIN. More than half needed HD (55%), all in the conventional modality. No patient was HD-dependent after hospital discharge.

The mean time for clinical and laboratory reassessment of patients (T1) was 16.3 ± 3.5 months, ranging from 11 to 23 months. The median of Cr at T1 was 1.19 (IQR: 0.95; 1.41 mg/dL) and the mean of eGFR 65.9 ± 21.7 mL/min/1.73m^2^, while the median of albuminuria was 8.36 (IQR: 6.0; 37 mg/g-Cr). On reassessment, hematuria (28.6%), microalbuminuria (30%), and CKD with eGFR < 60 mL/min/1.73 m^2^ (45%) were reported.

In the comparisons by need for HD, there were no difference regarding age, gender, presence of comorbidities, and length of hospital stay (Table 1). However, patients who required HD had higher levels of Cr and maximum urea on admission, and more pronounced reduction in eGFR (–19 [IQR: –39; –17] vs –5 [–10; 7] mL/min/1.73 m^2^, p = 0.004) and higher percentage of reduction (–28 [IQR: –43; –18] vs –4 [–14; 8] %, p = 0.004) between admission and reassessment periods (Table 1).

Mean T1 eGFR levels were lower compared to mean baseline eGFR (65.9 ± 21.7 vs 95.9 ± 16.7 mL/min/1.73 m^2^ p < 0.001), which is a reduction rate of –11.5 (IQR: –21; –2.1) ml/min/1.73 m^2^ per year. Comparing clinical and laboratory characteristics on T0 and T1 between patients with eGFR < 60 and eGFR > 60 on T1 (Table 2), mean age and length of hospital stay were higher in the group of patients with eGFR < 60 (p = 0.023 and p = 0.003, respectively). Furthermore, patients with eGFR < 60 at T1 had lower levels of eGFR on admission (p = 0.041). In addition, the group with eGFR < 60 at T1 showed higher levels of albuminuria and the presence of microalbuminuria (albuminuria > 30 mg/g-Cr) more frequently than the group with eGFR > 60 (p = 0.041). There was no difference when comparing hematuria variables at T1, and most hospitalization parameters, including DM, SAH, use of IMV, use of vasoactive amines, pulmonary involvement of COVID-19 on chest CT, need for HD, ferritin, and lymphocyte count between eGFR groups at T1 (Table 2).

**Table 2.** Comparison of characteristics between patients with eGFR < 60 and eGFR > 60 at T1

	eGFR ≥ 60 mL/min/1.73m^2^ (n = 11)	eGFR < 60 mL/min/1.73m^2^ (n = 9)	p
**Data at hospital admission (T0)**			
***Age, years***	57 ± 11	68 ± 8	0.023
***Female gender, n (%)***	5 (45.5)	5 (55.6)	1.000
***Hospitalization time, days***	31 (15; 46)	52 (45; 59)	0.003
***Comorbidities and treatments***			
Diabetes mellitus, n (%)	2 (18.2)	3 (33.3)	0.617
Arterial hypertension, n (%)	7 (63.6)	6 (66.7)	1.000
Use of ACE inhibitors or ARBs, n (%)	4 (36.4)	4 (44.4)	1.000
***Laboratory data and Kidney function***			
CRP on admission, mg/dL	4.1 (2.1; 20.4)	21.4 (14.6; 23.5)	0.08
Ferritin on admission, µg/L	781 (316; 1195)	1303 (917; 1579)	0.352
Lymphocyte count on admission, /mm^3^	1169 ± 454	1280 ± 461	0.597
Basal serum Creatinine, mg/dL	0.75 (0.61; 0.9)	0.8 (0.62; 0.89)	1.000
Basal eGFR, mL/min/1.73m^2^	99.09 ± 12.91	91.89 ± 20.59	0.352
Serum creatinine on admission, mg/dL	0.98 (0.61; 1.1)	1 (0.84; 1.15)	0.23
Blood Urea on admission, mg/dL	33 (28; 49)	33 (29; 62)	0.603
eGFR on admission, mL/min/1.73m^2^	91 ± 20	69 ± 23	0.041
AKIN, n (%)			0,226
*Stage 1*	2 (18.2)	0 (0)	
*Stage 2*	2 (18.2)	1 (11.1)	
*Stage 3*	7 (63.6)	8 (88.9)	
***Parameters during hospital stay***			
Maximum CRP, mg/dL	30.5 (19.5; 47.6)	36.9 (32.1; 40.1)	0.23
Maximum ferritin, µg/L	1650 (1504; 1934)	1650 (1381; 4224)	0.721
Minimum lymphocyte count, /mm^3^	505 ± 356	382 ± 184	0.362
Maximum serum creatinine, mg/dL	3.94 (1.73; 6.1)	3.84 (3.78; 6.14)	0.552
Maximum blood urea, mg/dL	159 (109; 239)	210 (153; 224)	0.656
Serum creatinine on hospital discharge, mg/dL	1.2 (0.7; 1.5)	1.4 (0.89; 2)	0.261
***Pulmonary function during hospital stay***			
Pulmonary involvement on CT, n (%)			0.234
*<25%*	5 (55.6)	1 (11.1)	
*25% – 50%*	1 (11.1)	2 (22.2)	
*50% – 75%*	2 (22.2)	4 (44.4)	
*>75%*	1 (11.1)	2 (22.2)	
Invasive ventilation, n (%)	7 (63.6)	8 (88.9)	0.319
Use of Vasopressor, n (%)	9 (81.8)	8 (88.9)	1.000
**Reassessment period (T1)**			
Time from discharge to follow-up, months	17 ± 4	16 ± 3	0.643
Systolic blood pressure, mmHg	146 ± 19	142 ± 15	0.653
Diastolic blood pressure, mmHg	92 ± 11	93 ± 21	0.878
Serum creatinine, mg/dL	1 (0.79; 1.25)	1.51 (1.2; 1.56)	0.006
eGFR, mL/min/1.73 m^2^	81 ± 17	48 ± 7	<0.001
Albuminuria, mg/g	6 (2.2; 11.2)	36.7 (7.6; 44.9)	0.041
eGFR reduction, mL/min/1.73m^2^	10 (–19; –1)	–18 (–39; –14)	0.201
eGFR reduction, %	–14 (–19; –1)	–31 (–43; –22)	0.067
eGFR reduction rate, mL/min/year	–14.4 (–24; –6.4)	–30 (–42.9; –27.7)	0.01
Microalbuminuria, n (%)	1 (9.1)	5 (62.5)	0.041
Hematuria, n (%)	1 (9.1)	3 (33.3)	0.285

ACEI: angiotensin-converting enzyme inhibitor, ARB: angiotensin receptor blocker, Cr: creatinine, eGFR: estimated glomerular filtration rate, AKIN: Acute Kidney Injury Network, Ur: urea, CRP: C-reactive protein, CT: computed tomography, DVA: vasoactive drug

**Figure 2.**
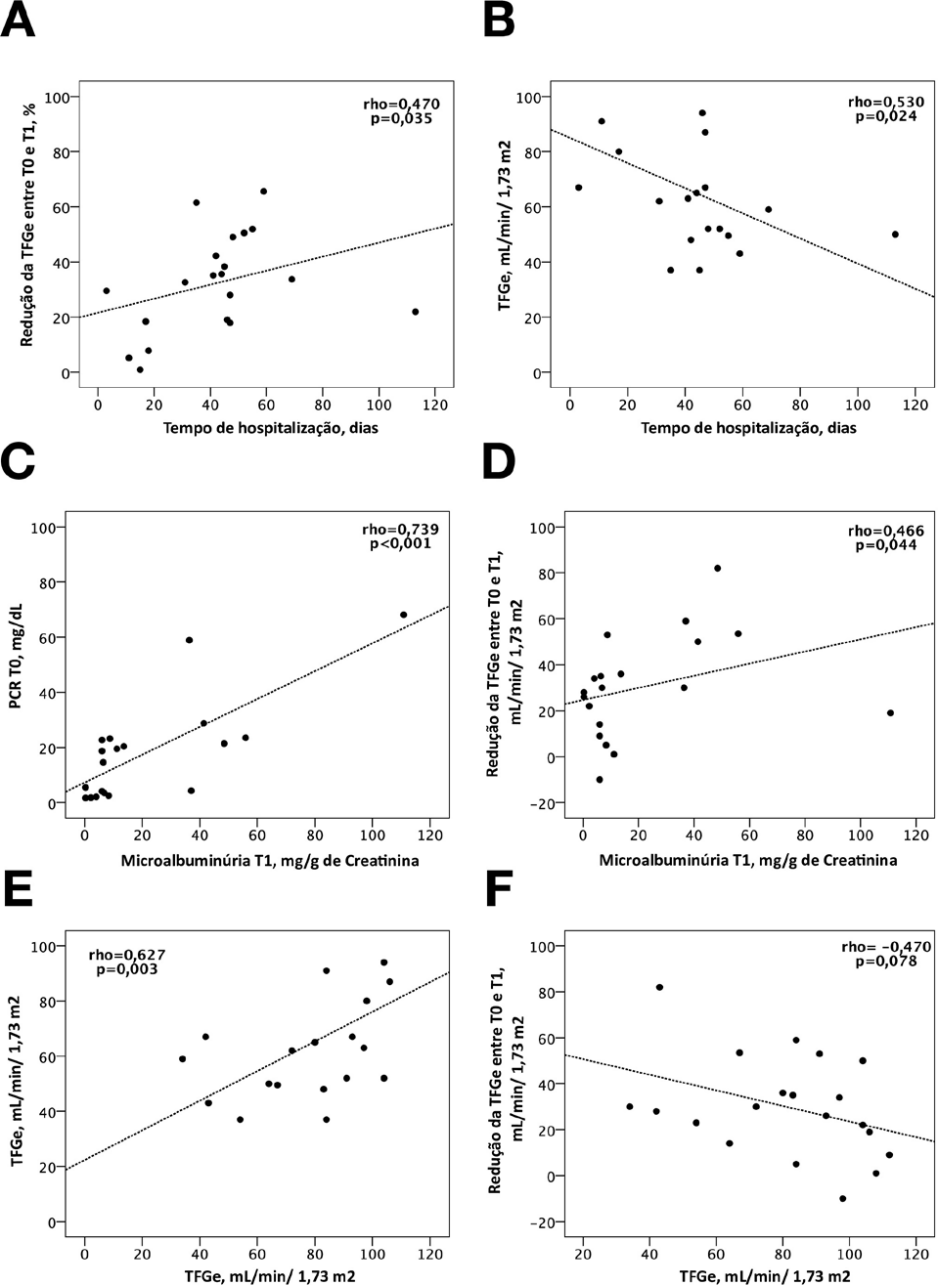
Correlations between eGFR at T1 and T0, eGFR reduction, hospitalization time, CRP, and microalbuminuria. A: Positive correlation between hospitalization time and percent eGFR reduction between T0 and T1. B: Negative correlation between hospitalization time and eGFR values at T1. C: Positive correlation between microalbuminuria values at T1 and CRP values at T0. D: Positive correlation between microalbuminuria values at T1 and reduction in eGFR between T0 and T1. E: Positive correlation between eGFR values at T0 and eGFR values at T1. F: Negative correlation between eGFR values at T0 and reduction in eGFR between T0 and T1.

Length of stay correlated negatively with eGFR values at T1 (rho = 0.530, p = 0.024) and positively with percentage of eGFR reduction between T0 and T1 (rho = 0.470, p = 0.035). Microalbuminuria at T1 was positively correlated with CRP values at T0 (rho = 0.739, p < 0.001) and with decrease in eGFR between T0 and T1 (rho = 0.466, p = 0.044). Moreover, eGFR on admission was positively correlated with eGFR at T1 (rho = 0.627, p = 0.003) (Figure 2).

## Discussion

There are few data on long-term progression to CKD in hospitalized patients who survived COVID-19-associated AKI. This is the first Brazilian study on the subject, and it showed a significant reduction in eGFR after about 16 months of hospitalization, on average by 32.2% compared to baseline eGFR. A Chinese study that evaluated AKI associated with COVID-19 found a 19.8% reduction in eGFR^9^. In AKI due to other causes, the reduction in eGFR is around 17%^10^.

In the present study, the length of hospital stay was negatively correlated with eGFR at T1 and the average number of days in hospital was higher in patients who evolved with eGFR < 60 mL/min. In contrast, a Swedish study with methodology similar to ours did not find differences in hospitalization time between those who evolved to CKD and those who did not^11^.

Older age is associated with a greater chance of failure to recover to baseline renal function after an AKI episode and consequent progression to CKD^12^. In this study, patients who evolved to CKD were older than patients who did not.

eGFR on admission was correlated with a better renal outcome at T1, in line with a study that compared the renal function of patients with AKI of various causes with the degree of impairment of eGFR on admission, determining the decline in eGFR in the long term^13^.

CRP is a risk factor for AKI associated with COVID-19^14,15^ and is also considered a predictor of worse clinical outcomes, such as severe AKI and slow renal recovery^14^. On the other hand, microalbuminuria is a well-established biomarker of progression to CKD^16^. In our study, there was a positive correlation between CRP levels at hospitalization and microalbuminuria levels at T1, reinforcing the inflammatory role in the progression to CKD. Furthermore, albuminuria levels also correlated positively with the drop in eGFR between T0 and T1.

There was no difference in the comparison between the other hospitalization variables in relation to the renal function assessment variables at T1. A similar study also found no association between these variables and progression to CKD, except for the need for HD and AKIN 3^11^.

A limitation of this study is the small sample size, which may have influenced the analysis of some variables and results. In addition, we recognize that the single measurement of eGFR can introduce bias during patient follow-up.

Patients with AKI associated with COVID-19 evolved with a significant reduction in eGFR after hospital discharge. Age, length of hospitalization, and need for HD were associated with a greater reduction in eGFR. Such results point to the need for routine nephrological evaluation in these patients.
